# Evaluation of guanidinoacetic acid supplementation on finishing beef steer growth performance, skeletal muscle cellular response, and carcass characteristics

**DOI:** 10.1093/jas/skae337

**Published:** 2024-11-02

**Authors:** Erika P Eckhardt, Wonseob Kim, Jerad Jaborek, Andrea J Garmyn, Donghun Kang, Jongkyoo Kim

**Affiliations:** Department of Animal Science, Michigan State University, East Lansing, MI 48824, USA; Department of Animal Science, Michigan State University, East Lansing, MI 48824, USA; Department of Animal Science, Michigan State University, East Lansing, MI 48824, USA; Department of Animal Science, Michigan State University, East Lansing, MI 48824, USA; Department of Food Science and Human Nutrition, Michigan State University, East Lansing, MI 48824, USA; Department of Animal Science, Michigan State University, East Lansing, MI 48824, USA; Department of Animal Science, Michigan State University, East Lansing, MI 48824, USA; Department of Food Science and Human Nutrition, Michigan State University, East Lansing, MI 48824, USA

**Keywords:** beef cattle, carcass characteristics, growth performance, guanidinoacetic acid, muscle growth

## Abstract

This study elucidated the effects of dosage-dependent guanidinoacetic acid (**GAA**) supplementation on growth performance, muscle responses, and carcass characteristics in finishing beef steers. Thirty crossbred Red Angus beef steers (395 ± 28.09 kg) were randomly assigned one of three treatments during a 146-d feedlot study: basal diet without GAA supplementation (**CONTROL**), 1g of GAA per 100 kg of body weight (**BW**) daily (**LOWGAA**), and 2 g of GAA per 100 kg of BW daily (**HIGHGAA**). Individual feed intake was monitored daily, growth performance parameters were collected every 28 d, and longissimus muscle (**LM**) biopsies occurred every 56 d. In biopsied LM, greater (*P* = 0.048) mRNA expression of *IGF-1* was observed in LOWGAA steers on day 112 compared to the CONTROL group. LOWGAA steers also exhibited greater expression of *myosin heavy chain* (***MHC***) *I* compared to CONTROL steers (*P* < 0.05) and *MHC IIA* compared to both CONTROL and HIGHGAA treatment groups (*P *< 0.01) on day 112. GAA supplementation resulted in no change in carcass characteristics, serum and LM tissue metabolites, LM composition, and Warner–Bratzler shear force values (*P *> 0.05). Data collected from this study demonstrate the influence of GAA supplementation on the gene expression of MHC isoforms and their role in skeletal muscle growth, differentiation, and muscle fiber-typing.

## Teaser

Our study ascertained the potential impact of guanidinoacetic acid (**GAA**) supplementation on skeletal muscle growth and differentiation, growth performance, and carcass characteristics in finishing beef steers. Our data highlighted the impact of GAA on myosin-heavy chain isoforms associated with animal metabolism and skeletal muscle growth and differentiation. Further explanation of the impact of GAA dosage-dependent supplementation over a specified duration in finishing beef steers is needed to elucidate cellular responses amid growth performance and carcass characteristic parameters.

## Introduction

Guanidinoacetic acid (GAA), also known as glycosamine, betacyamine, or N-amidinoglycine, serves as a precursor to creatine (Cr), a critical energy source for cells and tissues with high metabolic demands (Ostojic et al., 2020a; Ostojic, 2021). In the animal body, GAA is derived from both endogenous synthesis and dietary sources. The de novo synthesis of GAA involves a reaction between glycine and l-arginine, catalyzed by l-arginine: glycine amidinotransferase (AGAT). This process yields GAA and l-ornithine. Following synthesis in the kidneys, GAA is transported to the liver through the bloodstream, setting the stage for subsequent Cr production (Ostojic et al., 2020b). In the liver, and to a lesser extent in other organs such as skeletal muscle and the brain, GAA reacts with S-adenosyl-methionine (SAM) under the action of guanidinoacetate N-methyltransferase (GAMT), where GAA acquires a methyl group to form Cr, simultaneously releasing S-adenosyl-homocysteine (SAH). Creatine then enters cells through the sodium- and chloride-dependent creatine transporter 1 (SLC6A8 transporter 1), a Na+/Cl− dependent creatine co-transporter (Yan et al., 2021). Within cells, Cr and ATP undergo a reversible reaction catalyzed by creatine kinase, producing phosphocreatine (PCr) and adenosine diphosphate (ADP) for energy production. Approximately 1.7% of Cr and PCr are metabolized into creatinine, which serves as a key indicator of skeletal muscle degradation and kidney function, as it is filtered by the kidneys and excreted in urine (Cala et al., 2008; Curt et al., 2015). Creatine plays a vital role in supporting aerobic ATP production in mitochondria, which is essential for energy generation, and has been shown to influence skeletal muscle hypertrophy both directly and indirectly. In addition, ATP production extends to the regulation of protein synthesis through the mammalian target of rapamycin (mTOR) pathway, highlighting its significant role in muscular development and energy management (Wallimann et al., 1992; Farshidfar et al., 2017).

Due to its efficient conversion into Cr, GAA has been explored and employed as an alternative source of Cr in feed additives for meat animals. The U.S. Food and Drug Administration (**FDA**) has approved the use of GAA as a dietary supplement at a maximum of 0.12% of the complete diet fed to poultry per 21CFR573.496 (Food and Drug Administration: Dept. of Health and Human Services, 2021). The European Commission, the Panel on Additives and Products or Substances Used in Animal Feed (FEEDAP) has determined that feeding 1,200 mg of GAA/kg in complete feed is safe for feeder/finisher pigs and broiler chickens destined for the meat industry (FEEDAP et al., 2022). Baker (2009) and Ibrahim et al. (2019) denote GAA as chemically more stable and economically more feasible to use compared to Cr. However, Speer et al. (2019) report GAA as a nonruminally protected supplement, with approximately 50% degraded in the rumen.

The administration of GAA can increase circulating Cr concentrations in cattle and stimulate muscle growth, protein synthesis, and weight gain in poultry and swine (Ahmadipour et al., 2018; He et al., 2018; Lu et al., 2020; Ostojic et al., 2020b). Previous studies from outside of the U.S. have investigated the effect of supplemental GAA on beef cattle growth. These studies reported administration of GAA (0.6 to 0.9 g/kg DM) for 60 to 90 d increased BW (18 to 30 kg) and blood Cr in Angus bulls with an initial BW range 379 to 443 kg (Li et al., 2020; Liu et al., 2021a, 2021c). These researchers observed the effects of GAA impact on DMI, body weight (**BW**), ADG, FCR, and various serum metabolites. Yet, the molecular response of skeletal muscle remains relatively unexplored. Furthermore, data on the effects of GAA in beef cattle are limited by comparison to those conducted using swine and poultry models. We hypothesized that GAA supplementation will influence muscle growth mechanisms through the synthesis of Cr. Thus, the objectives of this study were to 1) elucidate the physiological responses and growth potential associated with GAA supplementation, and 2) evaluate the effects of GAA on skeletal muscle responses and meat quality.

## Materials and Methods

### Experimental design and dietary supplement

All procedures were conducted with the approval of the Michigan State University (**MSU**) Institutional Animal Care and Use Committee (IACUC; PROTO202200229).

Thirty predominantly Red Angus steers (BW 395 ± 28.09 kg, 2 animals per treatment were 25% Akaushi) participated in a 146-d finishing trial. All cattle were born and raised on pasture before beginning the finishing trial at the Michigan State University Upper Peninsula Research and Extension Center (**UPREC**) in Chatham, Michigan. Prior to the initiation of the finishing trial (day 68), steers were moved indoors into a group pen setting and fed a growing diet consisting of 29.5% barley, 17.5% DDGs, 4.0% molasses, 1.0% limestone, and 48.0% chopped grass hay on a DM basis. Steers were transitioned from the growing diet with 2 intermediate step-up diets that replaced a portion of the hay for barley until achieving the finishing diet composition at approximately 3-wk intervals.

Individual BW measurements were recorded consecutively on days −1 and 0 relative to the initiation of treatment and before morning feed delivery at approximately 0800 hours. Steers were then randomly allocated to indoor individual pens (2.74 × 3.05 m^2^) containing sawdust bedding, a minimum of 2 wk prior to the start of GAA supplementation. Each pen featured a feed bunk, and an automatic waterer positioned at the front. Following this, animals were randomly divided into 1 of 3 treatment groups as described (n = 10 per treatment): 1) Control (CONTROL), 2) 1 g GAA/100 kg BW (LOWGAA), 3) 2 g/100 kg BW (HIGHGAA). Since GAA has not been approved as a feed additive for beef cattle in the US, we secured authorization for its use as an Investigational Food Additive with a zero-day withdrawal period. This authorization, approval number B-013639-D-0002, was granted by the FDA for this trial.

The GAA (GuanAMINO, Evonik Ind, Essen, Germany) product comprises 96% feed-grade GAA and 4% cornstarch. Treatment concentrations were calculated using a rumen bypass rate of approximately 50% (Speer et al., 2019, 2020) and levels from previous beef cattle studies where supplementation dosage ranged from 0 g/kg to 1.6 g/kg DM and 0 to 0.4% DM intake (Li et al., 2020; Liu et al., 2021a). The maximum concentration approved by the FDA for feeding GAA to poultry is 0.12% of complete feed [21CFR573.496]. For blinding purposes in the study, CONTROL steers were given a bag containing 100 g of distiller’s dried grains with solubles (**DDGs**) without the GAA supplement. Individual treatment bags containing the DDGs, and the GAA (dependent on treatment) were fed each morning to their assigned steer 30 min before the delivery of morning feed. What treatment remained if any, was then placed on top of the delivered total mixed ration (**TMR**) to ensure supplement delivery.

The GAA quantity administered was updated monthly based on BW measurements collected every 28 d. All steers received the same finishing diet, which was fed using slick bunk management (Table 1). Within the established finishing ration, dairy monensin was added for additional Ca to balance the Ca:P ratio. Steers were treated for lice with a mixture of fossil shell flour (PERMA-GUARD Pure Diatomaceous Earth Products, Perma Guard In., Bountiful, UT) and sulfur powder commercial grade, consisting of 99+% purity (SULFUR POWDER, Duda Energy LLC, Decator, AL).

**Table 1.** Ingredient formulation and chemical composition of the experimental diet

Item	Finishing Diet
Ingredients, % DM	
Barley	63.40
DDGS	18.00
Chopped hay	11.90
Molasses	4.00
Limestone	0.80
Dairy monensin^1^	1.90
Analyzed composition ^2^	
DM, %	87.45
Crude protein, %	15.44
NDF, %	24.11
Crude fat, %	3.72
Ash	3.96
Ca, %	0.83
P, %	0.48
S, %	0.36
NE_m_, Mcal/kg	1.96
NE_g_, Mcal/kg	1.32

^1^Calculated analysis on a DM basis under manufacturer guidelines: Dairy Monensin (Nutra Blend LLC., Neosho, MO; 29.54 mg/kg) crude protein (0.9%), crude fat (0.3%), crude fiber (25%), Ca (20.4%). Ingredients listed as Rice Hulls, Calcium Carbonate, and Mineral Oil.

^2^The biweekly diet samples were analyzed at a commercial laboratory (DairyOne Forage Laboratory, Ithaca, NY).

### Feed sampling and BW measurement and analysis

Throughout the duration of the study, TMR feed samples were collected twice monthly and placed in a −20 °C freezer, with the freshest sample collected being sent off for wet chemistry analysis and all TMR samples submitted for dry matter analysis (DairyOne Forage Laboratory, Ithaca, NY). All feed ingredients and a TMR sample were evaluated for CP, NDF, crude fat, ash, NE_m_, NE_g_, calcium (Ca), phosphorus (P), sulfur (S), and DM (Table 1). BW was measured every 28 d on days −1, 0, 28, 56, 84, 112, and 140. A final weight was taken at the end of the trial before loading steers onto the trailer for trucking to the commercial abattoir located in Falmouth, MI.

### Blood serum collection and analysis

Blood samples were collected from the jugular vein of all steers (n = 30) on days 0, 28, 56, 84, 112, and 140 before feeding. Venipuncture blood was collected using an 18-gauge × 1.5 needle (VACUETTE^®^, Greiner Bio-One North America Inc., Monroe, NC) into serum tubes (evacuated BD Vacutainer^®^ Serum Tubes, Becton, Dickson, and Company, Franklin Lakes, NJ) for serum separation. All blood samples were placed on ice, transported to MSU, East Lansing Campus, and stored at 4 °C for 24 h. After which, serum was centrifuged at 2,500 × *g* for 15 min at 4 °C. The serum was aliquoted into sterile 1.5 mL tubes and stored at −20 °C until analysis.

The serum was analyzed for Cr, GAA, and creatinine. Samples were provided to the MSU Mass Spectrometry Core for analysis. About 100 µL of serum was mixed with 400 µL of extraction solvent (90:10 acetonitrile/water + 1 mM ammonium formate and 625 nM creatine-d3 as internal standard). Samples were further diluted 1:20 in 70:30 acetonitrile/water + 1 mM ammonium formate. Samples were analyzed using a Waters Xevo TQ-XS mass spectrometer with a Waters Acquity UPLC. Five µL of sample was injected onto a Waters Acquity Premier UPLC BEH-Amide column (2.1 × 100 mm) and compounds were separated using the following 10 min gradient: initial conditions were 5% mobile phase A (water + 10 mM ammonium formate + 10 mM ammonium hydroxide) and 95% mobile phase B (95:5 acetonitrile/water + 10 mM ammonium formate + 10 mM ammonium hydroxide), hold at 5% A until 1 min then ramp to 60% A at 6 min, hold at 60% A until 7 min, return to 5% A at 7.01 min and hold until 10 min. The column temperature was 40 °C and the flow rate was 0.3 mL/min. Compounds were ionized by electrospray ionization with a capillary voltage of 1 kV, source temp 150 °C, desolvation temp 400 °C, and desolvation gas and cone gas flows of 800 and 150 L/h, respectively. MS/MS parameters are listed in Supplementary Table 1. Data were processed using Masslynx software and compound amounts were calculated using creatine-d3 as the internal standard.

*Free amino acid collection and analysis*: 50 µL of serum was used from days 0, 56, and 112 for free amino acid analysis. Isolation prep was done at the MSU Muscle Biology lab and provided to the MSU Mass Spectrometry Core for analysis. Samples were mixed with 400 µL extraction solvent (water containing 13C, 15N-labeled amino acids internal standards) before being heated in a 90 °C water bath for 5 min before centrifugation. Centrifuge parameters were set at 4 °C with a run time of 10 min at 13,000 × *g*. The solution was then run through a 0.2 µM hydrophilic PTFE filter (Millipore Sigma, Burlington, MA), 100 µL of extracted sample was then mixed with 100 µL of 20 mM PFHA (Perfluoroheptanoic acid, Millipore Sigma) into LC autosampler vials (ThermoFisher Scientific, Waltham, MA) for analysis. Samples were analyzed using a Water Xevo TQ-S Micro UPLC/MS/MS (Waters Corp, Milford, MA). Ten µL of sample was injected onto a Waters Acquity HSS-T3 UPLC column (2.1 × 100 mm), and amino acids were separated using the following 13 min gradient: initial conditions were 100% mobile phase A (10 mM PFHA in water) and 0% mobile phase B (acetonitrile), hold for 1 min at 100% A then ramp to 65% B at 8 min, ramp to 90% B at 8.01 min and hold until 9 min, return to 100% A at 9.01 min and hold until 13 min. The column temperature was 40 °C and the flow rate was 0.3 mL/min. Compounds were ionized by electrospray operating in positive ion mode with a capillary voltage of 1.0 kV, source temp of 150 °C, desolvation temp of 350 °C, cone gas flow at 40 L/h and desolvation gas flow at 800 L/h. Multiple reaction monitoring parameters for MS/MS analysis are listed in Supplementary Table 2. The following Amino acids were analyzed: Ala, Arg, Asn, Asp, Glu, Gln, Gly, His, Ile, Leu, Lys, Met, Phe, Pro, Ser, Thr, Trp, Tyr, and Val.

### Longissimus muscle biopsy and analysis

Skeletal muscle biopsies were performed on the Longissimus muscle (**LM**) between the 12th and 13th ribs using a custom-made 6 mm Bergstrom biopsy needle, following a modified procedure (Smith et al., 2019) on days 0, 56, and 112. On day 0, 18 steers were sampled (n = 6 per treatment), and on days 56 and 112, 24 steers were sampled (n = 8 per treatment). Samples were taken from the same steers consistently through the duration of the study from the right side on days 0 and 112, careful to avoid the previous biopsy sites, and from the left side on day 56. The preparation of the area involved a sequence starting with hair removal, then initial cleaning using povidone–iodine for brushing, and then an antiseptic wash with 70% alcohol and povidone–iodine. Subsequently, the area was anesthetized using 15 mL of 2% Injectable Lidocaine (Covetrus, Portland, ME) for 10 min. A small incision was made with a scalpel, and approximately 0.5 g of muscle tissue was obtained using a biopsy needle. In order to minimize blood contamination in the muscle sample, efforts were made to retrieve the sample successfully on the first attempt. However, when insufficient muscle tissue was obtained, a second entry was made to secure an adequate sample and blotted to remove any blood remnants. The samples collected were instantly snap-frozen in liquid nitrogen and then transported on dry ice back to the MSU Muscle Biology Lab. Upon arrival, samples were promptly transferred to a −80 °C freezer for storage until further analysis.

### Carcass data collection

Following the 146-d finishing study, steers were transported 428 km to a federally inspected commercial abattoir (Falmouth, MI). Steers were held overnight at the abattoir and slaughtered the following morning under the United States Department of Agriculture (**USDA**) oversight for beef processing. After carcass chilling (48 h), trained personnel from MSU collected carcass data. The individual measurements for each carcass (n = 30) were hot carcass weight (**HCW**), fat depth between the 12th and 13th rib, ribeye area (REA), kidney, pelvic, and heart fat percentage (KPH%), marbling score, and USDA quality grade. USDA yield grade (USDA, 2017) was calculated from HCW, fat thickness, REA, and KPH using the following equation (USDA YG = 2.5 + ((2.5 × ((Preliminary Yield Grade − 2)/2.5) + (0.2 × (%KPH/100)) + (0.0038 × HCW) − (0.32 × REA (sq. in.)).

Instrumental color was obtained and averaged across 3 regions of the cut surface of the left LM after a minimum of 30 min of bloom time. The Commission on Illumination L* a* b* (CIE; Lightness, L*; redness, a*; yellowness, b*) color space values were measured using a Nix Pro 2 Color Sensor (Nix Sensor Ltd., Hamilton, Ontario, CA). CIELAB reference values were achieved at D50 illuminant and 2° observer angle. Ultimate pH was obtained 48 h postmortem from the left LM cut surface using a portable meat pH meter (Hanna Instruments, Smithfield, RI).

### Evaluation of muscle characteristics postmortem

After carcass data collection, an 8.89 cm portion of LM from the left side of the carcass was excised from the anterior end of the strip loin. These samples were placed in plastic bags and transported on wet ice to the MSU Meat Laboratory, where they were held at 3 °C until 3 d postmortem.

At 3 d postmortem, at least 200 g of muscle tissue from each carcass at the same anatomical location was retained for compositional analysis. The remaining loin sections were sliced into 2.54 cm steaks and randomly assigned to postmortem aging periods (day 3, 14, or 28 postmortem). Steaks were vacuum packaged and held at 2 to 4 °C during their respective aging period.

### Composition analysis

The 200 g samples allocated for compositional analysis from each carcass were cubed and then homogenized using a NutriBullet (Capital Brands Distribution LLC., Los Angeles, CA). Proximate analysis was conducted using an AOAC-approved near infrared spectrophotometer (FoodScan, FOSS NIRsystems, Inc., Laurel, MD) to determine fat, moisture, and protein percentage.

### Cooking loss and Warner–Bratzler shear force analysis

Once steaks reached their respective postmortem aging period (day 3, 14, or 28), steaks were cooked to determine cook loss and Warner–Bratzler shear force (**WBSF**) values. Steaks were weighed prior to cooking to assess cook loss. They were cooked on a George Foreman clamshell grill (Model GR390FP, Spectrum Brand Inc, Middleton, WI) until the internal temperature reached 70 °C. The temperature was monitored using a digital thermometer (Cooper-Atkins Platinum RTD Thermometer 36036). After achieving the target internal temperature, steaks were removed from the grill, allowed to rest for 2 min, and then reweighed. Steaks were placed on individual polystyrene foam plates, covered with polyvinyl chloride film, and stored at 3 °C for 24 h.

After cooling for 24 h, six 1.3-cm diameter cores were extracted from each steak parallel to the muscle fibers, using a drill-mounted corer. Cores were then individually sheared perpendicular to the muscle fibers using a TA-XT Texture Analyzer (Stable Micro System Ltd, Godalming, UK) equipped with a V-shaped Warner–Bratzler blade, operating at 200 mm/min crosshead speed.

### mRNA extraction and RT-qPCR analysis

Total RNA extraction from all biopsy samples was performed utilizing TRIzol reagent (ThermoFisher Scientific), as detailed previously (Kim et al., 2023; Smith et al., 2023). For each sample (0.1 g), 1 mL of cold TRIzol reagent was added to the tube along with two 5-mm sterilized stainless-steel beads and processed using the TissueLyser System II (QIAGEN, Hilden, Germany) and subjected to a frequency of 180 Hz for 2 min to facilitate rapid tissue breakdown. Following homogenization, the supernatant was transferred to a new tube, to which 200 µL of chloroform was added. The tubes were then vortexed and allowed to incubate at room temperature for 2 min. Following homogenization, the tubes were centrifuged at 12,000 × *g* for 10 min at 4 °C. Subsequently, the upper aqueous layer was transferred to a new tube. About 500 µL of isopropanol was added, followed by vortexing and a 15 min incubation at room temperature before centrifuging again at 12,000 × *g* for 15 min at 4 °C. The supernatant was then completely decanted, and the pellet was washed with 1 mL of 70% ethanol, vortexed, and centrifuged at 12,000 × *g* for 10 min at 4 °C. After discarding the supernatant, the RNA pellet was washed with 1 mL of 100% ethanol, vortexed, and centrifuged at 12,000 × *g* for 10 min at 4 °C. The supernatant was removed, and the tubes were left to air dry for 10 min. After drying, the RNA pellets were re-suspended in DEPC-treated water and vortexed. Subsequently, the samples were incubated at 60 °C for 10 min to facilitate complete dissolution. Following this incubation, the samples were vortexed again, briefly cooled, and centrifuged to collect any undissolved particles. The messenger ribonucleic acid (mRNA) concentration was determined using a NanoDrop One/OneC Microvolume UV-Vis Spectrophotometer (ThermoFisher Scientific). The RNA was then diluted to a concentration of 0.5 ng/µL with DEPC-treated water in preparation for cDNA synthesis. The removal of genomic DNA and the synthesis of cDNA were performed using the QuantiTect Reverse Transcription Kit (QIAGEN), adhering to the instructions provided by the manufacturer. Following synthesis, the cDNA concentration was diluted to 100 µg/µL and stored at −20 °C for future use.

Real-time quantitative polymerase chain reaction (RT-qPCR) analysis was conducted using an Applied Biosystems QuantStudio 6 Pro (ThermoFisher Scientific) to assess the expression levels of genes linked to muscle hypertrophy. The expression of housekeeping genes *ribosomal protein subunit 9 (RPS9)* and *hydroxymethylbilane synthase (HMBS)* was measured to normalize the gene expression data across samples. These housekeeping genes were selected for their stable expression across bovine tissues, ensuring accurate relative quantification of target genes (Bionaz and Loor, 2007; Janovick-Guretzky et al., 2007; Smith et al., 2019, 2023). Genes of interest within this study include *myogenin* (***MyoG***), *myogenic differentiation* (***MyoD***), *myogenic factor 5* (***Myf5***)*, insulin-like growth factor 1 (****IGF-1****), ribosomal protein S6 kinase B1* (***S6KB1***), *paired* box gene 7 (***Pax7***), and *myosin heavy chain (****MHC****) I, IIA,* and *IIX*. Relative quantification values of cDNA were performed using Applied Biosystems TaqMan Fast Advanced Master Mix (ThermoFisher Scientific) and TaqMan Gene Expression Assays (ThermoFisher Scientific; Table 2). Assays were conducted in duplicates as per thermal cycle parameters established by the manufacturer (45 cycles of 15 s at 95 °C and 1 min at 60 °C).

**Table 2.** The nucleotide sequences of PCR primers and probes for the Taqman assay

Gene^1^	TaqMan probe assay	Manufacturer
*RPS9*	Bt03272016_m1	Thermo Fisher
*HMBS*	Bt03234763_m1	
*MyoD*	Bt03244740_m1	
*MyoG*	Bt03258928_m1	
*IGF-1*	Bt03252282_m1	
*Myf5*	Bt03223134_m1	
*P70* ^*S6K*^	Bt00923436_m1	
*MHC Ⅰ*	Bt03224257_m1	
Gene	Primer and probe sequence (5ʹ to 3ʹ)	Thermo Fisher
*Pax7*		
Forward	GCCCTCAGTGAGTTCGATTAG	
Reverse	GATGCTGTGCTTGGCTTTC	
Probe	6FAM-TTCGTCCTCCTCCTCCTTCTTCCC-MGBNFQ	
*MHC ⅡA*		
Forward	GCAATGTGGAAACGATCTCTAAAGC	
Reverse	GCTGCTGCTCCTCCTCCTG	
Probe	6FAM-TCTGGAGGACCAAGTGAACGAGCTGA-TAMRA	
*MHC ⅡX*		
Forward	GGCCCACTTCTCCCTCATTC	
Reverse	CCGACCACCGTCTCATTCA	
Probe	6FAM-CGGGCACTGTGGACTACAACATTACT-TAMRA	

^1^ *RPS9*: ribosomal protein subunit 9, *HMBS*: hydroxymethylbilane synthase, *MyoD*: myogenic differentiation 1, *MyoG*: myogenin, *IGF-1*: insulin-like growth factor 1, *Myf5*: myogenic factor 5, *P70*^*S6K*^: ribosomal protein S6 kinase beta 1, *Pax7*: paired box gene 7, *MHC*: myosin heavy chain.

### Protein extraction and western blot analysis

LM biopsy tissue from day 112 timepoint, previously stored at −80 °C, underwent protein extraction and subsequent Western Blot (**WB**) analysis. Approximately 0.1 g of tissue was placed into a 2.0 mL microcentrifuge lock cap tube, along with two 5 mm stainless-steel TissueLyser beads (QIAGEN), and 500 µL of protein extraction reagent (T-PER; tissue protein extraction reagent, ThermoFisher Scientific) and protease inhibitor (Halt Protease Inhibitor Cocktail, EDTA-Free, ThermoFisher Scientific). Samples were run on TissueLyserII (QIAGEN) at a frequency of 180 Hz for 2 min to allow for adequate tissue breakdown. The aqueous phase (approximately 500 µL) was transferred to a new 1.5 mL snap-top tube and centrifuged at 10,000 × *g* for 5 min and 4 °C. The aqueous phase (300 µL) was removed, placed into a new 1.5 mL tube, and stored at −80 °C until further use. A standard curve was established using a bicinchoninic acid assay protein quantification kit (ThermoFisher Scientific). Tubes were incubated for 30 min at 37 °C. Tubes were then vortexed and centrifuged, and a NanoDrop One/One^C^ Microvolume UV-Vis Spectrophotometer (ThermoFisher Scientific), set at resonance of 562 nm, was used for quantification of protein concentration. Samples were diluted to 1 µg/µL and stored in a −80 °C freezer until WB analysis.

#### Light molecular weight (LMW) protein:

 Twenty nanograms of protein were added to 10 µL of sample buffer (Bolt LDS, ThermoFisher Scientific), 6 µL of deionized water, and 4 µL of samples reducing agent (Bolt, ThermoFisher Scientific) in a 1.5 mL tube and denatured for 10 min at 70 °C and 85 °C for 2 min. The electrophoresis was conducted using a running buffer solution composed of 20 × MES (2-N-morpholino ethane sulfonic acid) SDS (sodium dodecyl sulfate) running buffer (ThermoFisher Scientific), antioxidant (Bolt, ThermoFisher Scientific), and nanopore water. Polyacrylamide gels (12-well Bolt 4% to 12% Bis-Tris Plus, ThermoFisher Scientific) were prepped, and 5 µL of protein ladder (PageRule Plus Pre-stained Protein Ladder, ThermoFisher Scientific) was placed in the first and last well with 30 µL of denatured samples placed into subsequent individual wells within the gel and ran with a constant voltage (200 V, 20 m). At this point, gels were removed, rinsed with nanopure water, and transferred onto nitrocellulose membrane using iBlot 2 Dry Blotting System (ThermoFisher Scientific).

#### Heavy molecular weight (HMW) protein:

Fifteen nanograms of protein were added to 7.5 µL of sample buffer (NuPAGE LDS, ThermoFisher Scientific), 4.5 µL of deionized water, and 3 µL of samples reducing agent (NuPAGE, ThermoFisher Scientific) in a 1.5 mL tube and denatured for 10 min at 70 °C and 85 °C for 2 min. Running buffer solution (20 × SDS tris-acetate running buffer; NuPAGE, ThermoFisher Scientific), antioxidant (NuPAGE, ThermoFisher Scientific), and nanopure water were used for electrophoresis. Polyacrylamide gels (12-well NuPAGE Novex Tris-Acetate, ThermoFisher Scientific) were prepped, and 10 µL of protein standard (HiMark Pre-Stained heavy molecular weight (HMW), ThermoFisher Scientific) was placed in the first and last wells with 20 µL of denatured samples placed into subsequent individual wells within the gel and ran with a constant voltage (150 V, 50 m). Gels were removed, rinsed with nanopure water, and transferred onto nitrocellulose membrane using iBlot 2 Dry Blotting System (ThermoFisher Scientific).

Membranes of both HMW and LMW were blocked in a blocking solution containing nonfat dry milk powder (Research Products International, Mount Prospect, IL) and TBST ((TBS; BIO-RAD Laboratories, Hercules, CA) and Tween 20 (BIO-RAD Laboratories)) for 1 h at room temperature. Membranes were rinsed with 1 × TBST, 3 times for 5 min, and trimmed based on estimated molecular weight against the associated ladder. 1 × TBST with 5% bovine serum albumin (Fisher Scientific) was used for primary and secondary antibody dilution. Antibodies of interest for this study include primary antibodies: anti-mTOR, rabbit monoclonal, dilution of 1:1000 (Cell Signaling, Danvers, MA), anti-phospho-mTOR, rabbit polyclonal, dilution of 1:1000 (Cell Signaling), antiprotein kinase B (Akt), mouse monoclonal, dilution of 10:1000 (Developmental Studies Hybridoma Bank; DSHB, Iowa City, IA), anti-phospho-Akt, rabbit polyclonal, dilution of 1:1000 (Cell Signaling), anti-p70 ribosomal S6 kinase (p70^S6K^), rabbit polyclonal, dilution of 1:1000 (Cell Signaling), and anti-phospho-p70^S6K^, rabbit monoclonal, dilution of 1:1000 (Cell Signaling). Secondary antibodies include Goat antirabbit IgG H&L (HRP), rabbit polyclonal, dilution of 1:2000 (Abcam) and Goat antimouse IgG H&L (HRP), mouse polyclonal, dilution of 1:1000 (Abcam). Primary antibodies were incubated at 4 °C overnight and rinsed 3 times with 1 × TBST for 5 min. Secondary antibodies were added, incubated for 1 h, and rinsed following the previously mentioned steps. WB bands were observed by applying 2 mL of chemiluminescent substrate (SuperSignal West Pico, ThermoFisher Scientific). Signal detection was achieved through the ChemiDoc system (BIO-RAD Laboratories). Protein expression was normalized against housekeeping protein GAPDH, and signal intensity was calculated using ImageJ software (NIH, Bethesda, MD).

### CRE and phosphocreatine tissue analysis

Approximately 0.1 g of biopsied LM was separated from the initial sample collected on days 0, 56, and 112. Samples were weighted prior to submission to MSU Mass Spectrometry Core for analysis. Tissue analysis was conducted using 750 µL of extraction solvent (75:25 acetonitrile/water + 10 mM ammonium formate + 50 uM creatine-d3) and ground using a polypropylene pestle. Samples were kept at 4 °C for 4 h in solvent before being centrifuged, and the solution was transferred into a new tube and stored. The pellet from the initial extraction was then combined with 750 µL of extraction solvent, vortexed, and kept at 4 °C overnight. Both extraction supernatants were combined and diluted to 1:100 with a solution of 70:30 acetonitrile/water + 10 mM ammonium formate (no IS). Samples were analyzed using the same LC-MS/MS methods as for the serum samples outlined previously (Supplementary Table 1).

### Statistical analysis

A completely randomized design was implemented for the allocation of animals and subsequent analyses. Preliminary analysis was conducted using PROC MIXED SAS 9.4 (SAS Inst. Inc., Cary, NC) to evaluate whether initial BW was significant as a covariate in relation to growth performance, a nonsignificant covariate was obtained, therefore it was removed from the model. Thus, statistical and graphical representations of the data were performed using GraphPad Prism version 10.1.1 (Dotmatics, Boston, MA). In this setup, individual animals served as the experimental units. Growth performance, carcass characteristics, and shear force were assessed using a two-way ANOVA, with day being the repeated measure. The model used is defined: Y_klm_ = µ + T_k_ + D_l_ + T_k_D_l_ + S_m_ + e_klm_ where µ= overall mean, T_k_ = fixed effect of GAA, D_l_ = fixed effect of day, and T_k_D_l_ = fixed effect of the interaction between treatment × day, S_m_ = random effect of steer, and e_klm_ = residual error. Tukey’s post hoc test was also conducted.

Similarly to growth performance characteristics, day 0 was analyzed as a covariate for serum metabolites Cr, GAA, and Creatinine. Day 0 was found to be significant as a covariate and remained in the model. Serum metabolites were run using PROC MIXED in SAS 9.4, using the following model: Y_klm_ = µ + T_k_ + D_l_ + T_k_D_l_ + S_m_ + D_0_ + e_klm_ where µ= overall mean, T_k_ = fixed effect of GAA, D_l_ = fixed effect of day, and T_k_D_l_ = fixed effect of the interaction between treatment × day, S_m_ = random effect of steer, D_0_ = day 0 metabolites concentration as a covariate, and e_klm_ = residual error.

The mRNA gene expression and tissue Cr and PCr analysis had variable sample sizes between day 0 and later time points days 56 and 112 as outlined previously in the LM biopsy and analysis subsection of the materials and methods. Analysis was conducted utilizing a two-way ANOVA followed by Tukey’s post hoc test conducted in GraphPad with day being the repeated measure. The model used is defined: Y_klm_ = µ + T_k_ + D_l_ + T_k_D_l_ + S_m_ + e_klm_ where µ= overall mean, T_k_ = fixed effect of GAA, D_l_ = fixed effect of day, and T_k_D_l_ = fixed effect of the interaction between treatment × day, S_m_ = random effect of steer, and e_klm_ = residual error. Tukey’s post hoc test was also conducted. Data are presented as the mean ± SEM to accurately convey the variability within the data. The significance level was set at an ɑ of 0.05. Thus, a *P*-value of less than 0.05 was considered statistically significant, while *P*-values ranging from 0.05 to 0.10 were interpreted as tendencies.

## Results

### Growth performance

No differences were observed in the final BW (*P* = 0.93). Similarly, overall average daily gain (**ADG**; days 0 to 146) did not differ among treatments. However, a treatment × day interaction was exhibited (*P* = 0.04), with a tendency to increase ADG in the first 28 d of supplementation for HIGHGAA compared to CONTROL steers (*P* = 0.05). Overall, dry matter intake (**DMI**) was not affected by GAA treatment (*P* = 0.30). The feed-to-gain ratio (**F:G**) did not show any overall differences among the treatment groups (*P* = 0.22; Table 3).

**Table 3.** Growth performance of steers fed GAA for 146 d

	Treatments^1^			SEM^2^	*P*-value
Item	CONTROL	LOWGAA	HIGHGAA		
No. steers	10	10	10		
Initial BW^3^, kg	395.0	397.0	397.0	5.130	0.99
Final BW^3^, kg	603.0	601.0	601.0	6.588	0.93
ADG, kg					
Days 0 to 28	1.10	1.29	1.51	0.072	0.07
Days 29 to 56	1.68	1.56	1.67	0.062	0.72
Days 57 to 84	1.28	1.39	1.07	0.067	0.14
Days 85 to 112	1.45	1.39	1.38	0.052	0.83
Days 113 to 146	1.62	1.38	1.38	0.055	0.12
Overall, days 0 to 146^3^	1.42	1.39	1.40	0.027	0.91
DMI, kg					
Days 0 to 28	8.69	8.52	9.78	0.339	0.27
Days 29 to 56	9.46	9.19	10.68	0.289	0.10
Days 57 to 84	9.24	9.36	9.72	0.234	0.70
Days 85 to 112	10.06	10.00	10.25	0.195	0.87
Days 113 to 146	10.29	9.99	10.40	0.189	0.67
Overall, days 0 to 146^3^	9.55	9.41	10.17	0.119	0.30
F:G					
Days 0 to 28	7.62	7.24	6.93	0.385	0.78
Days 29 to 56	5.87	6.14	6.60	0.200	0.34
Days 57 to 84	7.57	7.52	9.83	0.600	0.20
Days 85 to 112	7.72	7.23	7.84	0.344	0.76
Days 113 to 146	6.53	7.46	7.89	0.329	0.23
Overall, days 0–146^3^	6.88	7.12	7.82	0.183	0.22

^1^Control: Steers fed a basal diet without guanicinoacetic acid (GAA) supplementation; LOWGAA group: Steers received 1 g of GAA per 100 kg of BW daily; HIGHGAA group: Steers received 2 g of GAA per 100 kg of BW daily; GAA was mixed with DDGs and fully consumed by the animals.

^2^Standard error of the mean.

^3^Reported as main effect.

### Carcass characteristics

Treatment had no effect on HCW (*P* = 0.97), REA (*P* = 0.34), adjusted backfat thickness (*P* = 0.51), and the percentage of KPH fat (*P* = 0.53; Table 4). Similarly, USDA yield grade (*P* = 0.40) and marbling score (*P* = 0.28) were not affected by treatment. The lightness (L*) and redness (a*) values remained similar, while a lesser yellowness (b*) value (*P *= 0.06) was observed in the HIGHGAA group compared to the CONTROL (Table 4). Ultimate pH did not show a difference due to treatment (*P* = 0.56).

**Table 4.** Carcass characteristics and meat quality of steers fed GAA for 146 d

	Treatments^1^			SEM^2^	*P*-value
Item	CONTROL	LOWGAA	HIGHGAA		
Hot carcass weight, kg	340.0	338.0	340.0	3.941	0.97
Adj. Backfat, cm	1.50	1.28	1.39	0.030	0.51
REA, cm^2^	80.84	82.13	77.35	0.210	0.34
KPH, %	2.66	2.45	2.33	0.080	0.53
USDA Yield grade	3.34	3.00	3.34	0.115	0.40
Marbling Score	499.0	556.0	576.0	20.214	0.28
Ultimate pH^3^	5.39	5.40	5.38	0.007	0.56
Lean color^4^					
L*	34.57	35.27	35.37	0.432	0.73
a*	20.27	20.10	19.97	0.266	0.90
b*	14.27	13.67	12.90	0.240	0.06

^1^Control: Steers fed a basal diet without guanicinoacetic acid (GAA) supplementation; LOWGAA group: Steers received 1 g of GAA per 100 kg of BW daily; HIGHGAA group: Steers received 2 g of GAA per 100 kg of BW daily; GAA was mixed with DDG’s and fully consumed by the animals.

^2^Standard error of the mean.

^3^Marbling score: 400 – 499 = small, 500 – 599 = modest.

^4^Ultimate pH collected 48 h postslaughter.

^5^Color analyzed 48 h postslaughter using Nix Sensor Ltd., Hamilton, Ontario, CA.

GAA supplementation had no impact on protein (*P *= 0.28), moisture (*P *= 0.25), and fat (*P *= 0.28) percentages of LM muscle (Table 5). Cook loss percentage did not differ in steaks aged 3 (*P* = 0.27), 14 (*P* = 0.52), or 28 (*P* = 0.90) d postmortem due to GAA supplementation. Similarly, the WBSF values did not differ among treatments after 3 (*P* = 0.19), 14 (*P* = 0.17), and 28 (*P* = 0.80) d postmortem wet agining.

**Table 5.** Chemical composition and texture analysis of LM of steers fed GAA for 146 d.

	Treatments^1^			SEM^2^	*P*-value
	CONTROL	LOWGAA	HIGHGAA		
Compositional analysis					
Moisture, %	71.84	70.87	70.41	0.357	0.254
Protein, %	21.36	21.07	20.80	0.141	0.281
Fat, %	6.46	7.62	8.23	0.453	0.275
Texture analysis					
Cook loss, %					
Day 3	16.69	14.74	14.82	0.549	0.270
Day 14	14.34	15.23	15.76	0.544	0.521
Day 28	17.04	17.50	17.64	0.594	0.897
WBSF, kg					
Day 3	4.24	4.10	3.58	0.156	0.192
Day 14	3.47	3.37	3.00	0.107	0.172
Day 28	3.09	3.21	3.09	0.081	0.801

^1^Control: Steers fed a basal diet without guanicinoacetic acid (GAA) supplementation; LOWGAA group: Steers received 1 g of GAA per 100 kg of BW daily; HIGHGAA group: Steers received 2 g of GAA per 100 kg of BW daily; GAA was mixed with DDGs and fully consumed by the animals.

^2^Standard error of the mean.

### Serum GAA, creatine, and creatinine level

Serum GAA metabolites did not alter with varying dosages in GAA supplementation (*P *> 0.05; Table 6). Over time, regardless of treatment, the serum GAA concentration gradually decreased as days on feed increased (*P* = 0.02). However, no changes (*P* = 0.58) in serum Cr levels were detected across different GAA supplementation concentrations. Similar to GAA, the overall concentration of serum Cr decreased over time (*P* = 0.03). Serum creatinine concentrations were not altered with varying GAA supplementation dosage (*P* > 0.05). Creatinine levels remained consistent as days on feed increased regardless of GAA supplementation (*P* > 0.05), with no treatment × day interaction observed (*P* > 0.05).

**Table 6.** Changes in serum metabolites of steers fed GAA for 146 d

	Treatment^1^				Day					*P*-value			
Sera analysis^3^	CON	LOW GAA	HIGH GAA	SEM^2^	28	56	84	112	140	SEM^2^	Day	Trt	Trt × day
Creatine, µM	191.40	179.55	191.73	9.433	200.17	199.90	186.10	176.33	175.30	8.369	0.03	0.58	0.46
GAA, µM	17.26	18.47	16.88	0.890	20.31	18.95	16.44	16.08	15.91	1.031	0.02	0.57	0.20
Creatinine, µM	85.13	96.53	93.26	5.127	88.83	97.44	88.88	90.64	92.41	4.301	0.44	0.35	0.65

^1^Control: steers fed a basal diet without guanicinoacetic acid (GAA) supplementation; LOWGAA group: steers received 1 g of GAA per 100 kg of BW daily; HIGHGAA group: steers received 2 g of GAA per 100 kg of BW daily; GAA was mixed with DDG’s and fully consumed by the animals.

^2^Standard error of the mean.

^3^Serum analysis conducted by MSU Mass Spectometry Core.

GAA supplementation at either 1 or 2 g/100 kg BW resulted in no changes to the concentration of free amino acids within the serum (*P* > 0.05) and no interaction between GAA and sample collection (*P* > 0.05; Table 7). However, Ala, Arg, Asn, Asp, Glu, Gly, His, Ile, Leu, Phe, Pro, Ser, Thr, Trp, Tyr, and Val exhibited changes in the concentration of serum over time (*P* < 0.05).

**Table 7.** Plasma amino acid levels in GAA-supplemented beef finishing steers

	Treatments^1^			SEM^2^	*P-*value
Item	CONTROL	LOWGAA	HIGHGAA		
*Amino acid* ^3^ *, nM*					
Asparagine					
Day 0	32.91	29.45	36.03	1.898	0.380
Day 56	24.38	30.15	22.62	1.536	0.109
Day 112	25.24	27.47	28.54	1.791	0.758
Aspartic acid					
Day 0	10.83	10.64	11.15	0.731	0.961
Day 56	8.21	8.40	8.10	0.468	0.966
Day 112	7.61	7.67	7.21	0.589	0.947
Glutamic acid					
Day 0	124.85	118.48	111.23	4.908	0.543
Day 56	87.04	85.97	78.05	3.087	0.448
Day 112	104.85	99.10	97.47	5.155	0.838
Glutamine					
Day 0	219.69	212.41	251.57	13.09	0.445
Day 56	213.16	233.08	215.40	8.627	0.603
Day 112	248.79	251.92	243.56	13.02	0.968
Glycine					
Day 0	280.86	251.00	287.19	15.824	0.624
Day 56	251.41	270.15	230.78	11.188	0.369
Day 112	225.35	223.58	197.55	12.415	0.609
Serine					
Day 0	110.40	104.49	113.45	4.314	0.706
Day 56	81.02	96.16	84.02	3.755	0.224
Day 112	91.63	100.85	93.55	4.988	0.742
Methionine					
Day 0	32.38	26.95	33.06	1.590	0.235
Day 56	26.21	28.84	26.74	1.235	0.666
Day 112	31.64	31.16	31.61	1.610	0.992
Proline					
Day 0	88.15	82.76	94.76	4.293	0.538
Day 56	66.22	76.69	75.48	3.223	0.363
Day 112	84.02	89.49	93.32	5.141	0.772
Arginine					
Day 0	189.12	186.06	208.83	7.685	0.437
Day 56	157.74	159.35	150.63	5.240	0.783
Day 112	163.70	182.57	162.31	7.516	0.485
Histidine					
Day 0	71.59	67.38	77.35	3.150	0.445
Day 56	75.14	80.73	72.98	2.636	0.480
Day 112	86.42	88.76	88.05	5.287	0.984
Lysine					
Day 0	146.70	130.82	150.79	6.110	0.384
Day 56	122.50	128.63	123.12	5.663	0.896
Day 112	144.48	144.49	132.22	7.198	0.738
Tryptophan					
Day 0	60.84	56.06	68.15	2.744	0.198
Day 56	58.36	61.43	65.40	2.287	0.467
Day 112	71.61	75.90	77.26	3.025	0.744
Tyrosine					
Day 0	71.05	64.33	78.63	2.872	0.125
Day 56	43.89	46.90	43.37	2.018	0.751
Day 112	50.13	56.58	53.26	2.918	0.683
Valine					
Day 0	238.24	213.77	254.33	11.137	0.338
Day 56	186.34	191.38	198.72	10.038	0.887
Day 112	228.51	245.17	244.63	13.553	0.859
Phenylalanine					
Day 0	72.93	67.53	72.89	2.706	0.661
Day 56	57.59	62.72	60.55	2.265	0.667
Day 112	71.12	82.60	77.82	3.941	0.506
Isoleucine					
Day 0	110.70	95.28	110.92	4.387	0.257
Day 56	77.73	85.15	80.22	3.666	0.716
Day 112	97.32	98.46	100.82	4.803	0.957
Leucine					
Day 0	218.02	187.08	229.94	10.044	0.203
Day 56	155.01	165.97	171.17	8.365	0.736
Day 112	199.21	207.56	212.30	10.297	0.879
Alanine					
Day 0	244.22	236.65	238.48	12.409	0.969
Day 56	193.91	215.80	190.00	8.029	0.381
Day 112	216.13	238.02	221.48	14.479	0.824
Threonine					
Day 0	83.53	68.89	91.62	4.787	0.145
Day 56	54.67	62.50	62.10	3.286	0.402
Day 112	66.91	69.62	77.19	4.197	0.601

^1^Control: Steers fed a basal diet without guanicinoacetic acid (GAA) supplementation; LOWGAA group: Steers received 1 g of GAA per 100 kg of BW daily; HIGHGAA group: Steers received 2 g of GAA per 100 kg of BW daily.

^2^Standard error of the mean.

^3^Amino acids extracted via 1x of ^13^C,^15^N stable isotope-labeled amino acid internal standards (MilliporeSigma).

### Skeletal muscle GAA, creatine, and creatinine level

**Figure 1.**
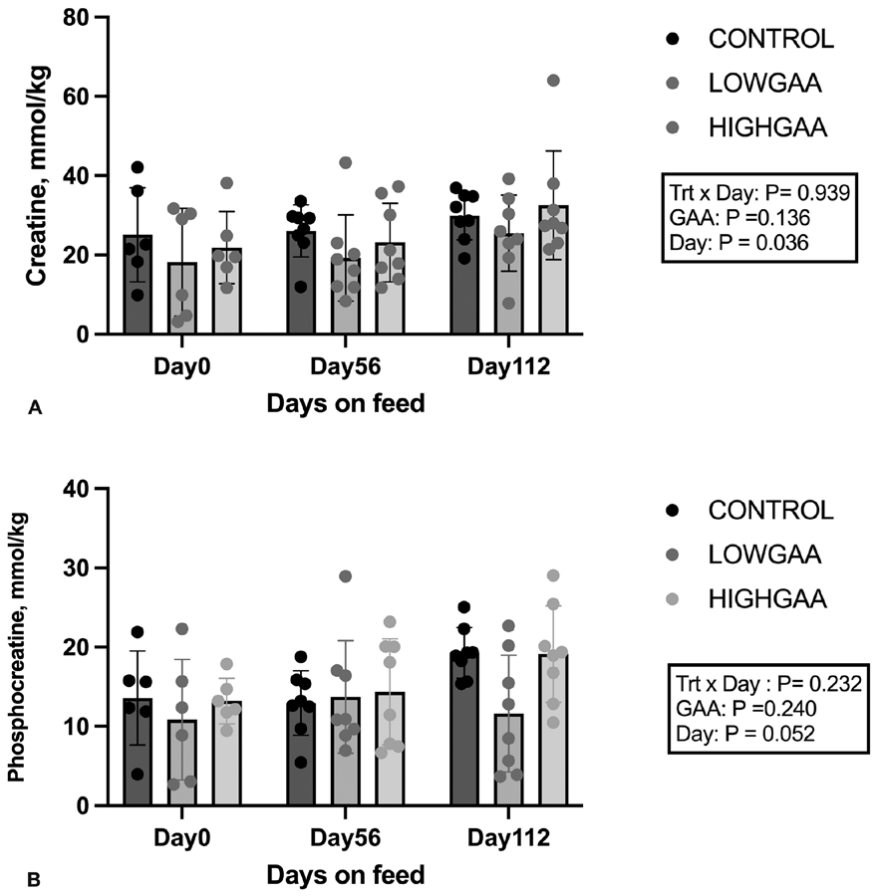
Changes in Longissimus dorsi metabolite concentration of creatine and phosphocreatinine in finishing beef steers supplemented with GAA. Control, LOWGAA: 1 g of GAA per 100 kg of BW daily. HIGHGAA: 2 g of GAA per 100 kg of BW daily. **1A**: creatine (mmol/kg), **1B**: phosphocreatine (mmol/kg). *Indicates significant differences in GAA supplementation (*P* < 0.05). Error bar represents SD.

Supplementation with GAA did not alter Cr (*P* = 0.14) and PCr (*P* = 0.24) concentrations in LM (Figure 1). However, the absolute amount of Cr (mmol/kg) was greater (*P* = 0.04) as the steers spent more time on feed, with a similar tendency observed for PCr (*P* = 0.05; Figure 1).

### Gene expression and WB analysis

**Figure 2.**
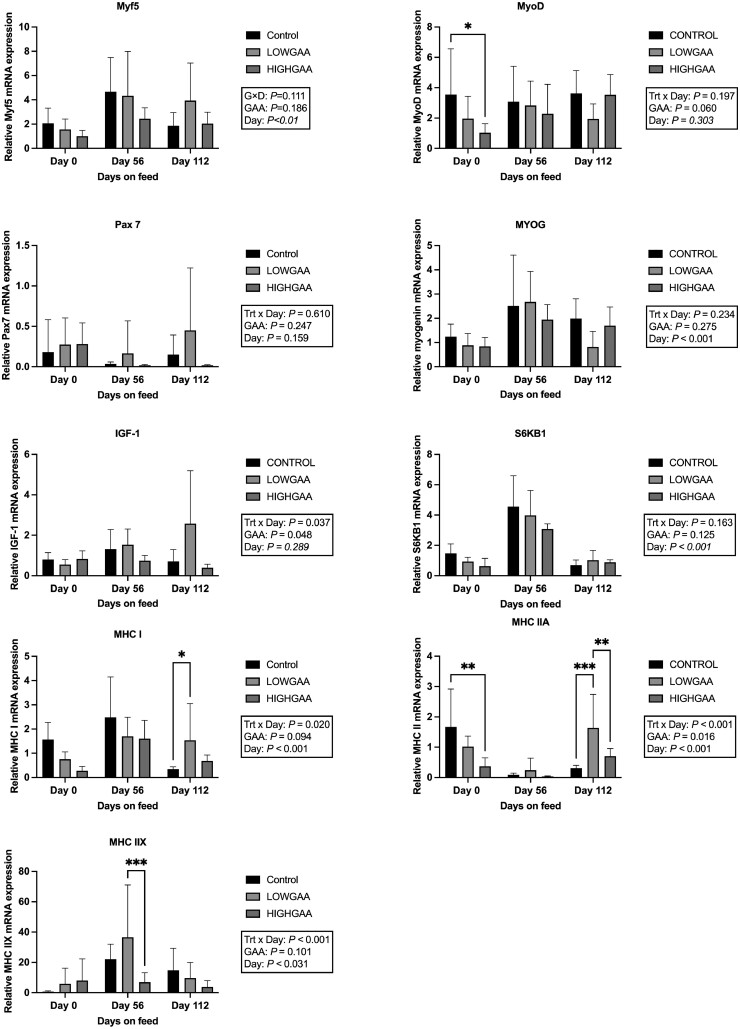
Changes in gene expression related to skeletal muscle growth in the Longissimus dorsi of beef steers following GAA supplementation. Control, LOWGAA: 1 g of GAA per 100 kg of BW daily. HIGHGAA: 2 g of GAA per 100 kg of BW daily. **2A:** myogenic factor 5 (*Myf5*), **2B:** myogenic differentietion 1 (*MyoD)*, **2C:** paired box gene 7 *(Pax7*), **2D:** myogenin (*MyoG*), **2E:** insulin-like growth factor 1 (*IGF-1*), **2F:** ribosomal protein S6 kinase beta 1(*P70*^*S6K*^), and *MHC* isoforms: **2G:***MHC 1*, **2H:***MHC IIA,* and ***3*I:***MHC IIX. P*-values determined by two-way ANOVA (* *P* < 0.05, ** *P* < 0.01, *** *P* < 0.001). Error bar represents SD.

Expression of *Myf5* (*P *= 0.19), *MyoG* (*P* = 0.28), and *Pax7* (*P *= 0.25) were not affected by GAA treatment. An effect of day was observed in *Myf5* expression (*P* < 0.01), with elevated expression levels on day 56 compared to day 0 (*P *< 0.001), and day 112 against day 0 (*P* = 0.047). *MyoG* expression exhibited a day effect (*P* < 0.001), elevated expression on day 56 compared to day 0 (*P* < 0.001) levels, and day 56 abundance compared to day 112 (*P* = 0.01). *MyoD* tended to alter expression among treatment (*P *= 0.06; Figure 2B) but had no day or treatment × day interaction (*P* > 0.05).

A day × treatment interaction effect was detected (*P* = 0.04) in *IGF-1* mRNA expression (Figure 2E); on day 112, *IGF-1* expression was greater in steers fed LOWGAA (2.57) compared to CONTROL (0.71; *P* = 0.003) and HIGHGAA (0.39; *P* > 0.001). *IGF-1* also exhibited a significant treatment effect (*P* = 0.048) in LOWGAA steers compared to their HIGHGAA counterparts. However, no treatment effects were observed (*P* = 0.163) in *S6kB1* mRNA expression, although a day effect was noted (*P* < 0.01), with expression being greater on day 56 compared to days 0 and 112.

**Figure 3.**
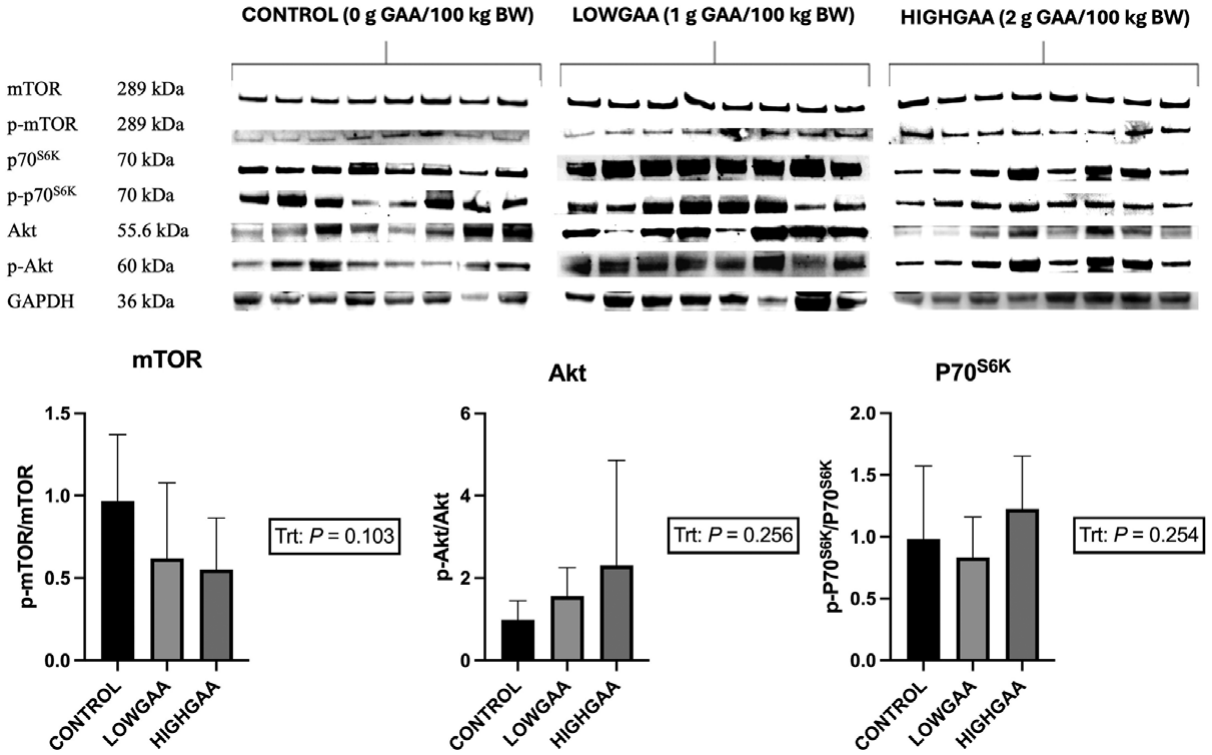
Alteration of protein concentration levels in Longissimus dorsi skeletal muscle at day 112 of GAA supplementation in finishing beef steers. Control, LOWGAA: 1 g of GAA per 100 kg of BW daily, HIGHGAA: 2 g of GAA per 100 kg BW daily. *P*-values determined by one-way ANOVA. * Indicates significant differences in main treatment effect (*P* < 0.05). Error bar represents SD.

An interaction between treatment and day was observed (*P* = 0.02) in *MHC I* mRNA expression, with a greater expression of *MHC I* in LOWGAA steers on day 112 compared to the CONTROL group (*P* = 0.03). A tendency (*P* = 0.09; Figure 2G) was exhibited for treatment impact on *MHC I* expression (*P* = 0.09). *MHC IIA* expression was greater in the CONTROL group compared to HIGHGAA on day 0 (*P *= 0.001). On day 112, *MHC IIA* gene expression was upregulated in LOWGAA compared to both CONTROL and HIGHGAA groups (*P *< 0.01; Figure 2H). There was an interaction between treatment and day (*P* < 0.01), CONTROL steers exhibited decreased expression of *MHC IIA* at days 0 (1.67) to 56 (0.09; *P *< 0.001) and days 0 (1.67) to 112 (0.31; *P* < 0.001). LOWGAA-supplemented group had decreased expression from days 0 (1.02) to 56 (0.24; *P* = 0.046) and elevated expression levels at days 56 (0.24) to 112 (1.64; *P* < 0.001). Protein concentrations in the LM of the analyzed day 112 samples were not different (*P* > 0.05) for mTOR, Akt, and p70^S6K^ (Figure 3) in regard to treatment.

## Discussion

### Growth performance

Across the entire 146-d study, ADG did not differ for CONTROL, LOWGAA, and HIGHGAA steers (1.42, 1.39, and 1.40 kg, respectively). With a significant treatment × day interaction, GAA had a tendency to impact ADG in the first 28 d. ADG was greater by 0.22 kg/d for HIGHGAA steers compared to LOWGAA-supplemented counterparts in the first 28 d of the feeding period and 0.41 kg/d for HIGHGAA in contrast to CONTROL steers. It is important to note here that steers were well adjusted to their housing conditions and began their finishing diet at the start of the GAA supplementation. Previously, GAA supplementation in beef cattle has shown an improvement in ADG of 0.14 to 0.39 kg/d (Li et al., 2020, 2021; Liu et al., 2021c; Yi et al., 2024a) when supplemented GAA at 0.6 to 1.6 g/kg DM and 0.05 to 0.4% of GAA as a percentage of dietary DM. Furthermore, the method of supplementation and the energy density of the TMRs provided across research groups and the subsequent duration of supplementation. It is the fluctuations in low vs. high energy dense diets across the previously conducted studies that highlights the need to understand how density of diet fed can impact GAA effectiveness.

Previous research on the effects of GAA supplementation on DMI in cattle has yielded mixed outcomes. A Li et al. (2020) study reported greater DMI due to GAA supplementation, while others have found no significant difference (Liu et al., 2021c; Yi et al., 2024a), and another has observed a lesser DMI (Speer et al., 2020). Feed efficiency was also noted to improve with supplementation of GAA at 0.6 to 0.9, and 1.6 g/kg GAA on a DM basis and 0.05% to 0.2% GAA as a percentage of dietary DM (Li et al., 2020, 2021; Liu et al., 2021a, 2021c; Yi et al., 2024a). In other species, more notable changes were expressed for growth parameters including improved growth performance, feed efficiency, muscle creatine, and phosphocreatine levels for swine (Li et al., 2018; Lu et al., 2020; Valini et al., 2020). Poultry exhibited improved BW and weight gain, ADG, feed efficiency (Michiels et al., 2012; Córdova-Noboa et al., 2018; Ibrahim et al., 2019; Raei et al., 2020; Khalil et al., 2021), and sheep exhibited improved carcass characteristics, increased plasma hepatic GAA levels, and skeletal muscle phosphocreatine levels (Liu et al., 2021b; Ren et al., 2022; Zhang et al., 2022).

With no differences in growth performance observed, this potentially brings to question the internal level of de novo synthesis with each individual animal throughout days on feed compared to the levels of GAA used in this study estimated to surpass rumen viability. With methyl capabilities of the steers not measured in the samples we collected, approximately 50% rumen bypass was assumed based on Speer et al. (2019) and Speer et al. (2020). This brings the potential discussion for the rumen bypass rate of GAA when mixed or top-dressed on a TMR or against rumen and abomasal infusions and against the methyl group donation for enabling GAA conversion to Cr (Ostojic, 2016, 2021; Ostojic et al., 2020b). The direct top-dress has the potential to flood the system and when the established threshold has been achieved can excrete the excess GAA and Cr. In the instance of our steers, urine collection was not feasible and since serum metabolites were 24 h from the previous day’s supplementation window, this leads us to not be able to speculate on the instances of Cr turnover.

### Carcass characteristics

There has been limited research into the effects of GAA supplementation on meat quality in beef cattle (Li et al., 2021; Yi et al., 2024b). GAA supplementation did not impact carcass characteristics, meat composition, L*, a*, ultimate pH, cook loss, or WBSF. The lack of change in HCW is consistent with the findings from Yi et al., (2024b) in which GAA at 1.0 g/kg DM and GAA and methionine both at 1.0 g/kg DM saw no change in HCW, cold carcass weight, and other slaughter performance characteristics. A tendency to alter the b* color value was found due to GAA supplementation. The correlation between the yellowness in beef and its quality remains incomplete in our understanding. These findings are in contrast with Li et al. (2021) in which GAA supplementation in *Jinjiang* bulls caused lesser L* color values, drip loss percentages, and greater a* and b* color values. Yi et al. (2024b) also in contrast to our results found that GAA and GAA + methionine decreased L*, drip loss, cook loss, moisture percentage, and increased a*, eye muscle area, and crude protein percentage. There is a notable gap in literature addressing the impacts of GAA on traditional carcass characteristics, including backfat thickness, KPH%, YG, and marbling scores in beef cattle. In poultry, the supplementation of GAA at 1.0 and 1.5 g/kg has been shown to enhance carcass and breast yield while reducing abdominal fat deposition (Ahmadipour et al., 2018). Lu et al. (2020) observed that supplementation of 0.06% GAA increased the loin muscle area by 16.40 cm² in swine. Further emphasized, swine receiving 0.06% GAA supplementation exhibited decreased backfat thickness, drip loss percentage, and muscle fiber density while increasing shear force, muscle fiber cross-sectional area, and a tendency to increase IMF (Lu et al., 2020).

### Serum GAA, creatine, and creatinine levels

Dietary supplementation of GAA resulted in no difference of measured serum metabolites, these findings differ from those established in other cattle breeds supplemented with GAA at varying dosages, durations, and manners of administration (Ardalan et al., 2015; Li et al., 2020, 2021; Speer et al., 2020; Liu et al., 2021c; Yi et al., 2024a). It is critical to assess the timing of the last GAA feeding with the moment of serum collection from the subjects. Ostojic et al. (2014) reported temporal changes in GAA, Cr, and creatinine concentrations in humans postconsumption, noting that concentrations normalized to baseline within 24 h. Notably, the peak concentrations of both GAA and Cr occurred approximately 1 h after ingestion. Conversely, creatinine levels peaked 2 h post-ingestion and returned to their minimum 6 h after intake before ultimately returning to baseline concentrations. In our study, blood samples were drawn before the morning feeding roughly 24 h after the previous feeding. It is probable that serum GAA and Cr concentrations had returned to baseline in the present study.

Free amino acid levels within the serum were not different across treatments. Amino acids, upon consumption, either undergo protein synthesis, or when in excess undergo catabolism and are broken down as an energy source and undergo lipogenesis for fat storage. While no current research was found in beef cattle on the rate of absorption and time postconsumption, plasma amino acid concentrations spiked, leading to the potential probability of blood amino acid levels returning to baseline by the time serum samples were collected 24 h postconsumption. However, amino acid concentrations were altered across time for Ala, Arg, Asn, Asp, Glu, Gly, His, Ile, Leu, Phe, Pro, Ser, Thr, Trp, Tyr, and Val. Ala, Arg, Asn, Asp, Glu, Ile, Leu, Phe, Pro, Ser, Thr, Tyr, and Val decreased in concentration from days 0 to 56, with Asn, Asp, Glu, Ser, and Tyr also decreasing in concentration from days 0 to 112. Gly concentration only decreased from days 0 to 56 and days 0 to 112, and Glu decreased from days 56 to 112. This is similar to what was reported by Flynn et al. (2000), in that suckling piglets demonstrated lesser plasma amino acid concentrations of Ala, Arg, Glu, Ile, Leu, and Val with increased age. In our study, His and Trp concentration was increased from days 0 to 112, with His, Ile, Phe, Pro, Tyr, and Val increasing in concentration from days 56 to 112.

### Skeletal muscle GAA, creatine, and creatinine levels

The unaltered concentration of both Cr and PCr across treatments, with undetectable values of GAA and creatinine within our LM samples, varies from previous studies conducted in multiple species. Greater Cr concentration in muscle was noted for Angus bulls (Liu et al., 2021c), poultry at varying GAA-supplemented concentrations (Ringel et al., 2007), lambs (Ren et al., 2022), and swine (Li et al., 2018). Serum metabolites within a few hours post-GAA consumption reached peak concentration, further research is needed to better understand skeletal muscle metabolite’s concentration curve post-GAA supplementation to understand peak levels within tissue. To better understand how sampling duration postconsumption could play a critical role in understanding conversion time, peak concentration, and the rate of time it takes to return to baseline further needs to be established. Furthermore, Cr is the first energy source utilized in skeletal muscle. Although efforts were made to minimize stress while collecting skeletal muscle biopsies, the steers experienced some stress and may have utilized stored creatine during the LM biopsy collection process.

### Factors related to skeletal muscle growth

The anabolic effects of Cr on skeletal muscle hypertrophy have been extensively investigated across various species, with evidence suggesting that Cr supplementation activates genes associated with skeletal muscle growth. The direct impact of GAA on skeletal muscle growth appears limited; however, its efficacy improves when GAA is converted into Cr. Louis et al. (2004) reported that Cr-treated C2C12 myoblasts exhibited overexpression of IGF-1 and muscle regulatory factors (**MRFs**). In humans, when supplemented alongside heavy resistance training, Cr upregulated *myogenin* and *MRF-4* expression (Willoughby and Rosene, 2003). To date, no study has evaluated the response of MRFs to GAA feeding in beef cattle. In our current research, we detected no significant alterations in MRFs that matched the magnitude of changes observed in in vitro studies.

Supplementation of GAA increased *IGF-1* in the LM for LOWGAA compared to CONTROL and HIGHGAA steers, demonstrating the impact potential of GAA on skeletal muscle protein synthesis through driving anabolic and catabolic pathways. The altering of gene expression towards the end of the study forecasts its potential as a nonconventional growth technology in late-stage finishing steers in altering protein synthesis, or the duration of effectiveness ramps up with increased supplementation over time. In contrast, *IGF-1* expression in the LM was not altered for finishing swine (Lu et al., 2020). Liu et al. (2021c) noted GAA supplementation increased hepatic gene expression of *IGF-1, PI3K*, *Akt1*, and *mTOR* increased in bulls supplemented with 0.6 g/kg of GAA. Pigs supplemented with GAA had a higher hepatic expression of *IGF-1, PI3K*, *Akt1*, and *mTOR* (Lu et al., 2020).

In our study, dosage-dependent supplementation of GAA did not affect protein regulation in LM skeletal muscle on day 112. Conversely, in a growing lamb study, a diet with 0.09% GAA-supplemented diet resulted in greater concentrations of phosphorylated mTOR, Akt, 4EBP1, and S6K proteins (Li et al., 2022). Wang et al. (2018), noted that miR-133a-3p and miR-1a-3p genes in the C2C12 cells of mice supplemented with dosage-dependent GAA resulted in stimulation of the mTOR/Akt/S6K signaling pathway. Further studies in bullfrogs (Lin et al., 2021) and nursery piglets (Valini et al., 2020) noted upregulation and a tendency to upregulate, respectively, the mTOR signaling pathway due to varying GAA supplemental concentrations.

Steers supplemented with LOWGAA compared to CONTROL steers exhibited greater *MHC I* and *IIA* mRNA expression, which is associated with smaller muscle fiber diameters. However, *MHC I* and *IIA* expression in LOWGAA was not able to be correlated to potential tenderness characteristics across measured wet aging periods.

## Conclusion

The results of our study indicate the potential of GAA as a dietary supplement for promoting skeletal muscle growth in finishing beef steers. Supplementation with 1 g GAA per 100 kg BW increased GAA and creatinine levels at specific time points, though it did not affect overall circulating blood serum or muscle Cr levels. Additionally, GAA supplementation did not impact the overall growth performance and carcass traits. The influence of GAA on late-stage LM gene expression, particularly for *IGF-1* and *MHC IIA*, suggests that GAA may affect skeletal muscle fiber type, potentially improving meat quality and color. GAA supplementation also showed a tendency to have lesser b* lean color values. The future role of GAA in beef cattle supplementation warrants further research to determine the bypass rate and optimal dosage range of GAA for ruminant animals. Our current study suggests that 2 g of GAA per 100kg of BW when top-dressed is seemingly insufficient to enhance skeletal muscle growth in finishing cattle. Additionally, various scenarios involving methyl group donors should be considered.

## Supplementary Material

skae337_suppl_Supplementary_Tables

## Abbreviations

ADP: adenosine diphosphate
AGAT: l-arginine: glycine amidinotransferase
Akt: protein kinase B
BW: body weight
CF: crude fat
CK: creatine kinase
Cr: creatine
DDGS: distiller’s dried grains with soluble
DMI: dry matter intake
F:G: feed:gain ratio
FDA: U.S. Food and Drug Administration
FEEDAP: Panel on Additives and Products or Substances Used in Animal Feed
GAA: guanidinoacetic acid
GAMT: guanidinoacetate N-methyltransferase
HCW: hot carcass weight
HMBS: hydroxymethylbilane synthase
HMW: heavy molecular weight protein
IFA: Investigational Food Additive
IGF-1: insulin-like growth factor-1
KPH%: kidney, pelvic, and heart fat percentage
LM: Longissimus muscle
LMW: light molecular weight protein
Met: methionine
MHC: myosin heavy chain
mRNA: messenger ribonucleic acid
MSU: Michigan State University
mTOR: mammalian target of rapamycin
Myf5: myogenic factor 5
MyoD: myogenic differentiation
MyoG: myogenin
p70^S6K^: p70 ribosomal S6 kinase
Pax7: paired box gene 7
PCr: phosphocreatine
REA: ribeye area
RPS9: ribosomal protein subunit 9
RT-qPCR: real-time quantitative polymerase chain reaction
S6KB1: ribosomal protein S6 kinase B1
SAH: S-adenosyl-homocysteine
SAM: S-adenosyl-methionine
SDS: sodium dodecyl sulfate
SLC6A68: sodium- and chloride-dependent creatine transporter 1
TMR: total mixed ration
UPREC: Michigan State University Upper Peninsula Research and Extension Center
USDA: United States Department of Agriculture
WB: western blot
WBSF: Warner–Bratzler shear force
