# Supplementary material for: Evaluation of guanidinoacetic acid supplementation on finishing beef steer growth performance, skeletal muscle cellular response, and carcass characteristics
Source: J Anim Sci. 2024 Nov 2;102:skae337. doi: 10.1093/jas/skae337 (PMC11633455; doi:10.1093/jas/skae337)
Supplement: skae337_suppl_Supplementary_Tables [file skae337_suppl_supplementary_tables.docx]

**Table S1.** MS/MS parameters

| **Compound** | **Ionization mode** | **Parent ion** | **Daughter ion** | **Cone voltage** | **Collision energy** |
| --- | --- | --- | --- | --- | --- |
| Creatine | + | 131.95 | 89.96 | 28 | 10 |
| Creatine-d3 | + | 134.95 | 92.96 | 28 | 10 |
| Guanidinoacetatic acid | + | 117.99 | 75.94 | 28 | 10 |
| Creatinine | + | 113.99 | 85.95 | 20 | 25 |
| Phosphocreatine | - | 209.90 | 78.70 | 28 | 22 |

**Table S2.**  Amino acid MS/MS parameters

| **Compound** | **Parent ion** | **Daughter ion** | **Cone voltage** | **Collision voltage** |
| --- | --- | --- | --- | --- |
| Glycine | 76 | 30 | 17 | 8 |
| [^13^C_2_,^15^N]-Glycine | 79 | 32 | 17 | 8 |
| Alanine | 90.1 | 44 | 35 | 17 |
| [^13^C_3_,^15^N]-Alanine | 94.1 | 47.1 | 17 | 8 |
| Serine | 106.1 | 60 | 30 | 10 |
| [^13^C_3_,^15^N]-Serine | 110.1 | 63 | 19 | 10 |
| Threonine | 120.1 | 74 | 19 | 8 |
| [^13^C_4_,^15^N]-Threonine | 125.1 | 78.1 | 19 | 8 |
| Cysteine | 122 | 76 | 18 | 15 |
| [^13^C_3_,^15^N]-Cysteine | 126 | 79 | 18 | 15 |
| Asparagine | 133.1 | 74 | 35 | 14 |
| Aspartic Acid | 134.1 | 74 | 35 | 10 |
| [^13^C_4_,^15^N]-Aspartic Acid | 139.1 | 77 | 19 | 11 |
| Glutamine | 147.1 | 84 | 35 | 14 |
| Glutamic Acid | 148.1 | 84 | 34 | 14 |
| [^13^C_5_,^15^N]-Glutamine | 154.1 | 89.1 | 17 | 14 |
| Proline | 116 | 70 | 35 | 10 |
| [^13^C_5_,^15^N]-Proline | 122.1 | 75.1 | 35 | 10 |
| Valine | 118.1 | 72 | 35 | 9 |
| [^13^C_5_,^15^N]-Valine | 124.1 | 77.1 | 35 | 9 |
| Methionine | 150.1 | 104 | 19 | 9 |
| [^13^C_5_,^15^N]-Methionine | 156.1 | 109.1 | 19 | 9 |
| Tyrosine | 182.1 | 136.1 | 20 | 12 |
| [^13^C_9_,^15^N]-Tyrosine | 192.1 | 145.1 | 20 | 12 |
| Isoleucine and Leucine | 132.1 | 86 | 35 | 9 |
| [^13^C_5_,^15^N]-Leucine | 139.1 | 92 | 35 | 9 |
| Lysine | 147.1 | 84 | 19 | 14 |
| [^13^C_6_,^15^N_2_]-Lysine | 155.1 | 90.1 | 19 | 14 |
| Histidine | 156.1 | 110 | 20 | 12 |
| [^13^C_6_,^15^N_3_]-Histidine | 165.1 | 118.1 | 20 | 12 |
| Phenylalanine | 166.1 | 120 | 20 | 10 |
| [^13^C_9_,^15^N]-Phenylalanine | 176.1 | 129.1 | 20 | 10 |
| Arginine | 175.1 | 70 | 24 | 18 |
| [^13^C_6_,^15^N_4_]-Arginine | 185.1 | 75 | 24 | 18 |
| Tryptophan | 205.1 | 146 | 19 | 14 |
| [^13^C_11_,^15^N_2_]-Tryptophan | 218.1 | 156 | 19 | 14 |
